# Analysis of kidney dysfunction in orthopaedic patients

**DOI:** 10.1186/1471-2369-13-101

**Published:** 2012-09-03

**Authors:** Konstantinos Kateros, Christos Doulgerakis, Spyridon P Galanakos, Vasileios I Sakellariou, Stamatios A Papadakis, George A Macheras

**Affiliations:** 1 Department of Trauma and Orthopaedics, General Hospital of Levadia, Levadia, Greece

**Keywords:** Kidney dysfunction (KD), Orthopaedic population, Surgical procedure

## Abstract

**Backround:**

This retrospective study was undertaken to determine the incidence of kidney dysfunction (KD) and to identify potential risk factors contributing to development of KD in orthopaedic population following an elective or emergency surgery.

**Methods:**

A total of 1025 patients were admitted in our institution over a period of one year with various indications. Eight hundred and ninety-three patients (87.1%) had a surgical procedure. There were 42 (52.5%) male and 38 (47.5%) female with a mean age of 72 years (range: 47 to 87 years). We evaluated the following potential risk factors: age, comorbidities, shock, hypotension, heart failure, medications (antibiotics, NSAIDs, opiates), rhabdomyolysis, imaging contrast agents and pre-existing KD.

**Results:**

The overall incidence of KD was 8.9%. Sixty-eight patients developed acute renal injury (AKI) and 12 patients developed acute on chronic kidney disease (CKD). In sixty-six (82.5%) patients renal function was reversed to initial preoperative status. Perioperative dehydration (p = 0.002), history of diabetes mellitus (p = 0.003), pre-existing KD (p = 0.004), perioperative shock (p = 0.021) and administration of non-steroid anti-inflammatory drugs (NSAIDs) (p = 0.028) or nephrotoxic antibiotics (p = 0.037) were statistically significantly correlated with the development of postoperative KD and failure to gain the preoperative renal function.

**Conclusion:**

We conclude that every patient with risk factor for postoperative KD should be under closed evaluation and monitoring.

## Background

The overall incidence of kidney dysfunction (KD) after elective or emergency orthopedic surgical procedures is reported to reach 9.1% [1-6]. The risk of acute kidney injury (AKI) in surgical patients has been estimated to be approximately 1% of all hospitalized patients. It is known to be an independent predictor of poor in-hospital outcome [7-9].

However, certain patient populations are at much higher risk. Factors that are identified to increase the risk of AKI include age, past history of kidney disease, left ventricular ejection fraction less than 35%, cardiac index less than 1.7 L/min/m2, hypertension, peripheral vascular disease, diabetes mellitus, emergency surgery, and type of surgery [10].

Surgical procedures that are at higher risk for development of AKI include those involving coronary artery by-pass, cardiac valve reconstruction, open aortic aneurysm bypass, and liver transplantation [11-13]. Patients that undergo major orthopaedic procedures are also at high risk for KD due to the potential high volume of blood loss, severe electrolyte disturbances, development of perioperative infection or sepsis, and presence of several comorbidities that may impair renal function (i.e. diabetes, heart failure, severe arrhythmias, pulmonary embolism etc.) [1,14]. In addition, pre- or post-operative KD is a risk factor for postoperative complications, including acute renal failure and cardiovascular disease, leading to increased mortality and morbidity [15,16].

There are limited data on how to assess postoperative KD following major orthopaedic procedures. Our hypothesis is that several factors associated with these procedures such as the increased surgical time, the need for blood transfusion, and the use of perioperative antibiotics and analgesics may contribute to the development of acute kidney injury [1,2]. The purpose of this retrospective study is to determine the incidence of AKI following an orthopaedic surgery (elective or emergency) and to identify and the preoperative risks factors for the development of postoperative KD.

## Methods

We retrospectively analyzed the medical records of 1025 admissions to our institution in a period of 13 months between February 2008 and March 2009. The study protocol was approved by the Scientific Council and the Ethics Committee of Levadia General Hospital, Greece. Patients that underwent an elective or emergency orthopaedic procedure were eligible for inclusion to the study. Patients that were admitted for conservative treatment were excluded. There were 586 male and 439 female with a mean age of 64 years (range: 27 to 98 years, SD: ± 10.2). Demographics, indications for admission, comorbidities and length of stay in hospital (hospitalization) were assessed and all collected data are presented in Table 1.

**Table 1:** Demographics, indications for admission, comorbidities and length of stay in hospital (hospitalization) of all patients who admitted in our institution

**Gender**		**Co-morbidities (N = 697, 68%)**	
Male	586 (57.1%)		
Female	439 (42.8%)	Dehydration	187 (26.8%)
		Heart failure	153 (22%)
**Age (y), (mean, range)**		Diabetes mellitus	146 (21%)
	64 (27–98)	Arterial hypertension	74 (10.6%)
		Chronic obstructive airways disease	61 (8.7%)
**Admission category**		Cerebrovascular event	39 (5.6%)
Trauma	788 (76.8%)	Peripheral vascular disease	21 (3%)
Elective surgery	105 (10.2%)	Rheumatoid disease	10 (1.4%)
Other	132 (12.8%)	Parkinson’s disease	4 (0.5%)
		Malignancy	2 (0.2%)
**History of KD**			
Yes	46 (4.4%)	**Length of stay in hospital (mean, range, SD)**	9.5 (5–27 ± 4.2)
No	979 (95.5%)		

KD: kidney dysfuction, SD: Standard deviation.

All patients received medical intervention according to a pre-determined protocol. This involved a medical and social history and a physical examination. Investigations included full blood count, urine analysis, glucose, urea, creatinine, electrolytes, electrocardiogram, chest X-ray and other appropriate radiographs of musculoskeletal system.

The patients who developed KD [acute or acute on chronic kidney dysfunction (CKD)] post-operatively, were evaluated by a senior nephrologist and were classified according to the final outcome of renal function into (i) complete resolution, (ii) failure to return to prior kidney function, (iii) end-stage renal disease and (iv) death. Potential risk factors for KD were pulled from medical charts including age, perioperative hypotension or shock, and presence of comorbidities such as diabetes mellitus, coronary heart disease, heart failure, and CKD. The type and duration of use of potential nephrotoxic agents (i.e. contrast mediums, angiotensin converting enzyme inhibitors (ACE-I), angiotensin-II receptor antagonists (AT-II), diuretics, antibiotics (aminoglycosides), non-steroidal anti-inflammatory (NSAID) and opiates) were also recorded.

All cases with documented perioperative complications were prospectively followed-up in order to reveal potential correlations between the development of KD and associated comorbidities. KD according to Acute Kidney Injury Network (AKIN) criteria has been defined as the increase in serum creatinine ≥ 0.3 mg/dL or increase to ≥150% (1.5- to 3-fold) from baseline [17,18].

Also the outcome regarding the possibility to return to prior renal function was estimated. As “full recovery of renal function” was defined as the return of lab values to normal. “Incomplete recovery” was defined the improvement of renal function but still remaining over 120% of initial values. “Stable” was defined as no recovery and “Progression of renal dysfunction” as defined by an increase in Crea or decrease in GFR values comparing to the baseline (corresponding values at admission).

### Statistical Analysis

Data is expressed as mean ± standard deviation (S.D.) or median (in case of violation of normality) for continuous variables and as percentages for categorical data. The Kolmogorov - Smirnov test was utilized for normality analysis of the parameters. Univariate analyses were made by using the chi-square test or alternatively the Fisher exact test to analyze the relation between the outcome variable and the qualitative variables, whereas the Student t-test or Mann–Whitney U-test and One-way ANOVA or Kruskal - Wallis were used to analyze the relation between the outcome variable and the quantitative measures respectively.

All the estimated potential risk factors, such as the age, the comorbidities (diabetes mellitus), shock, hypotension, heart failure, medications (antibiotics, NSAIDs, opiates), rhabdomyolysis, imaging contrast agents and pre-existing KD, whether or not they demonstrated significant associations with outcome variable (renal filtration function) in univariate analysis were included in the model, and stepwise elimination (Wald method) was used to arrive at the final model. Goodness of fit was evaluated using the Hosmer-Lemeshow statistic.

A p-value of <0.05 (two sided) was used to denote statistical significance, though associations reaching borderline significance (0.05 < p < 0.1) were also identified as being of potential interest. All analyses were carried out using the statistical package SPSS vr15.00 (Statistical Package for the Social Sciences, SPSS Inc, Chicago, IL, USA).

## Results

From February 2008 to March 2009 in a total of one thousand and twenty-five patients which admitted in our institution, eight hundred and ninety-three patients (87.1%) had a major orthopaedic procedure. Specifically, there were 663 cases with lower extremities fractures, 124 cases with upper extremities fractures, 1 case with compartment syndrome, 56 cases with Total Hip Arthroplasty and 49 cases with Total Knee Arthroplasty. Of them, four hundred and sixty-six (52.2%) patients had numerous pre-existing comorbidities including, diabetes mellitus in 122 cases, heart failure in 76 cases, chronic obstructive airways disease in 20, and pre-existing CKD in sixteen cases. One hundred and five patients received Nephrotoxic agents, whereas dehydration at the time of admission was noted in 137 cases. Perioperatively 5 cases developed shock (3 hypovolemic and 2 cardiogenic) and one procedure was complicated with rhabdomyolysis after a severe crash injury of the lower extremity. Demographic data and perioperative complications of all surgical patients are summarized in Table 2.

**Table 2:** Characteristics of all the trauma patients and complications during the hospitalization

**Gender**		**Complications (N = 85. 9.5%)**			
Male / Female	468 (52.4%) / 425 (47.6%)			*pre-op*	*post-op*
		Urinary tract infection	29 (3.2%)	13	16
**Age (y), (mean, range)**	68 (36–93)	Acute myocardial infarction	12 (1.3%)	5	7
		Pneumonia	9 (1%)	4	5
**Indications for surgery**	893	Deep wound infection	8 (0.9%)	0	8
Pertrochanteric femoral fractures	317 (35.4%)	Deep vein thrombosis	8 (0.9%)	1	7
Femoral neck fractures	278 (31.1%)	Cerebrovascular accident	7 (0.8%)	2	5
Other fractures of lower extremities	68 (7.6%)	Death	5 (0.5%)	3	2
Upper extremities fractures	124 (13.8%)	Pulmonary embolism	3 (0.3%)	0	3
Compartment syndrome of tibia	1 (0.1%)	Gastrointestinal bleeding	3 (0.3%)	2	1
THA / TKA	56 (6.2%) / 49 (5.4%)	Rhabdomyolysis	1 (0.1%)	0	1

THA: Total Hip Arthroplasty, TKA: Total Knee Arthroplasty.

KD occurred in 80 (8.9%) cases. There were 42 (52.5%) male and 38 (47.5%) female with a mean age of 72 years (range: 47 to 87 years, SD: ± 9.9). 68 patients of 80 developed AKI and 12 patients developed acute on CKD. The demographic data and medical characteristics of patients who developed KD are shown in Table 3.

**Table 3:** Demographics and medical characteristics of patients who developed KD

**Gender**		**Aetiology of KD (N = 80)**	
Male	42 (52.5%)	Dehydration	13 (16.2%)
Female	38 (47.5%)	Diabetes mellitus	11 (13.7%)
		Shock	10 (12.5%)
**Age (y), (mean, range, SD)**	72 (47–87, ± 9.9)	Heart failure	2 (2.5%)
		Medications	
		NSAIDs	9 (11.2%)
		Antibiotics	6 (7.5%)
**Charasteristics of KD**		ACE-I	4 (5%)
		Diuretics	3 (3.7%)
Acute	64 (80%)	AT-II	3 (3.7%)
Acute on CKD	16 (20%)	Opiates	1 (1.2%)
		Rhabdomyolysis	1 (1.2%)
		Iodinated contrast agents	1 (1.2%)
		Pre-existing KD	16 (20%)

ACE-I: angiotensin converting enzyme inhibitors, AT-II: angiotensin-II receptor antagonists, NSAIDs: non-steroidal anti-inflammatory drugs, KD: kidney dysfunction, CKD: Chronic kidney disease.

Eight patients (10%) with KD had a low serum urea concentration (<17 mg/dL), at the time of KD diagnosis, 34 (42.5%) had a normal concentration (normal values: 17–52 mg/dL) and 38 patients (47.5%) had a raised serum urea concentration (>52 mg/dL). Seventeen (21.2%) had a low serum creatinine concentration (<0.7 mg/dL), forty-three (53.7%) had a normal creatinine (0.7-1.30 mg/dL) and 20 patients (25%) had a raised creatinine value. Mean GFR was 81,71 mL/min (ranging from 23 to 343 mL/min).

Sixty-six (82.5%) of 80 patients, recovered their renal function prior to surgery (Table 4). The mean length of stay in hospital of AKI patients increased by 29% compared to non-KD patients (9.8 days compared to 7.6 days, p < 0.001).

**Table 4:** Final outcome in patients with risk factors for KD

	**Good outcome**	**Poor outcome**	***p-value***
Dehydration (N = 13)	12 (92.3%)	1 (7.7%)	0.002
Diabetes mellitus (N = 11)	10 (91%)	1 (9%)	0.003
Shock (N = 10)	8 (80%)	2 (20%)	0.021
Heart failure (N = 2)	1 (50%)	1 (50%)	0.128
NSAIDs (N = 9)	8 (89%)	1 (11%)	0.028
Antibiotics (N = 6)	5 (88.3%)	1 (16.7%)	0.037
Opiates (N = 1)	1 (100%)	‐	N/A
ACE-I (N = 4)	2 (50%)	2 (50%)	0.128
Diuretics (N = 3)	2 (67%)	1 (33%)	0.079
AT-II (N = 3)	2 (67%)	1 (33%)	0.079
Rhabdomyolysis (N = 1)	‐	1 (100%)	N/A
Iodinated contrast agents (N = 1)	1 (100%)	‐	N/A
Pre-existing KD (N = 16)	14 (87.5%)	2 (12.5%)	0.004
Total (N = 80)	66 (82.5%)	14 (17.5%)	<0.0001

ACE-I: angiotensin converting enzyme inhibitors, AT-II: angiotensin-II receptor antagonists, NSAIDs: non-steroidal anti-inflammatory drugs, KD: kidney dysfuction, N/A: not applicable.

There was not statistical significant difference in mortality in patients with sustained AKI compared with patients with transient AKI. There was a statistically significant correlation of perioperative dehydration (p = 0.002), history of diabetes mellitus (p = 0.003), pre-existing renal dysfunction (p = 0.004), perioperative shock (p = 0.021) and administration of non-steroid anti-inflammatory drugs (NSAIDs) (p = 0.028) or nephrotoxic antibiotics (p = 0.037) with the development of irreversible renal filtration. On the other hand, heart failure, and administration of ACE-I, AT-II and diuretics were not statistically significantly correlated with perioperative KD. The sample size was found small and inappropriate for any correlation between the use of opiates, iodinated contrast agents or the presence of rhabdomyolysis and the development of KD.

Furthermore, we found a significant main effect of the estimated potential risk factors in renal function after an episode of AKI. A patient with diabetes mellitus has an 18.2-fold risk of irreversible KD, comparing with a patient without the disease (p = 0.003). Patient with perioperative shock of any etiology have 8.5 times greater risk for KD (p = 0.02). Also, there is an 8.5% higher risk of KD with an increase of age by one year (p = 0.05).

Thirty-nine (48.7%) patients showed full recovery of their renal function, 19 (23.8%) incomplete recovery, 15 (18.7%) were stable and 7 (8.8%) patients showed a progression of renal dysfunction. Three cases (21.4%) from the group with pre-existed KD (n = 14) developed ESRF (two with pre-existing KD and one with diabetes mellitus), while one patient (7.1%) died.

## Discussion

This study demonstrates an incidence of 8.9% of KD in a group of patients that had undergone major orthopaedic procedures in our hospital over a period of one year. Also an assessment of numerous of potential risk factors of postoperative KD was carried out.

Definition of acute renal failure is still controversial and sometimes confusing [19]. Authors have used terms such as renal insufficiency, renal dysfunction, acute renal failure (ARF), and renal failure requiring dialysis somewhat interchangeably [20]. The lack of consensus in the quantitative definition of ARF, in particular, has hindered clinical research since it confounds comparisons between studies. Some definitions employed in clinical studies have been extremely complex with graded increments in serum creatinine for different baseline serum creatinine values [7,21]. However, the Acute Dialysis Quality Initiative (ADQI) recognizing the need for a uniform definition for ARF, proposed a consensus graded definition, called the RIFLE criteria [22]. The ESRD is a term that only indicates chronic treatment by Kidney Injury Network, which included the ADQI group as well as representatives from other nephrology and intensive care societies. In addition, the term acute kidney injury (AKI) was proposed to represent the entire spectrum of acute renal failure [23-25].

Chronic kidney disease (CKD) as defined in 2002 by the Kidney Disease Outcomes Quality Initiative (K/DOQI) of the National Kidney Foundation is either kidney damage or decreased kidney function for 3 or more months [24]. Numerous reports in the literature attempt to assess various factors those contribute to postoperative outcome of kidney function. These factors can be described as the preoperative comorbid status, the type of surgical procedure, and the postoperative period [25-27].

It is known that the overall incidence of KD after elective or emergency orthopedic surgical procedure is up to 9.1% [1-6]. Moreover, it has been reported that pre-operative renal dysfunction is a risk factor for postoperative complications, including AKI and cardiovascular disease [15], leading to prolonged morbidity or hospital stay [28] and to increased mortality [16]. In addition, electrolyte and fluid balance have been implicated as important factors that can influence morbidity and complications [29,30]. We did not found a significant difference in mortality in patients with sustained AKI compared with patients with transient AKI. A possible explanation could be the small number of deaths so our findings should be regarded with caution.

It is generally recognized that risk factors for the development of KD in this population include dehydration, increasing age, diabetes mellitus, shock, heart failure, medications (NSAIDs, aminoglycosides, ACE-I, diuretics, AT-II, opiates), rhabdomyolysis, iodinated contrast agents and pre-existing KD. In a recent study by Bennet et al. [2], authors found that the male sex, vascular disease, hypertension, diabetes, chronic kidney disease and pre-morbid use of nephrotoxic medications were the main risk factors of AKI in patients with a fractured neck of femur. The most important risk factor for postoperative KD in the present series was the dehydration at admission; the second most significant was the history of diabetes mellitus while as third significant risk factor the pre-existing KD was noted. These findings may be associated with the increased mean age of KD group of patients. There are studies that support our findings [2,3]. A diminished physiological response to the trauma coupled with enforced fasting, anaesthetic agents and blood loss caused by the injury and surgery exacerbate this as an additional stress on an already frail patient. A recent analysis by White et al. [1], has shown that approximately one-third of patients presenting for surgical fixation of fractured neck of femur have at least moderate renal dysfunction on admission to hospital, a prevalence that increases with age. The authors indicated that these patients had a higher perioperative risk of opioid-induced respiratory depression, necessitating consideration of opioid-reducing strategies such as early fracture fixation, regular simple analgesia and regional nerve blockade. On the contrary, our study was not able to estimate the opiates as a significant risk factor for KD, due to limited sample size. On the other hand we found that there is an 8.5% higher risk of KD with an increase of age by one year. Novis et al. [31] reviewed the potential preoperative risk factors for postoperative KD and showed that cardiac risk factors appeared predictive of postoperative renal failure. In our study, heart failure was a potential risk factor for the development of postoperative KD, but it was not correlated with the recovery of kidney function at the preoperative level. In this series potential risk factors for the development of KD were pre-existing KD, dehydration, diabetes mellitus, shock, NSAIDs, aminoglycosides, ACE-I, AT-II, diuretics, heart failure, opiates, rhabdomyolysis and iodinated contrast agents.

Optimal fluid balance, as well vigilance and caution especially for patients with high risk of KD, are crucial. This statement is in agreement with other authors [1,2,28,31]. White et al. [1] suggest that preoperative kidney dysfunction should alert the anesthetists for the appropriate adjustment of peri-operative analgesic administration, and adequate intra-operative intravenous fluid management, in patients with ‘borderline’ renal function. Multitrauma cases or elderly with hip fractures frequently present dehydrated at admission. A diminished physiological response to the trauma coupled with enforced fasting, anaesthetic agents and blood loss caused by the injury and surgery exacerbate this [2]. Absolute or relative hypovolaemia is a significant risk factor for ARD development [21]. Numerous of studies have shown that post-operative outcome can be positively influenced by optimising volume status and oxygen delivery perioperatively using invasive monitoring [32-34].

Measures to prevent preoperative KD include close evaluation of patients with risk factors and monitoring by a specialist. A consideration of opioid-reducing strategies such as early fracture fixation, regular simple analgesia (but not non-steroidal anti-inflammatory drugs) and regional nerve blockade is necessary. Also an appropriate volume of fluid was required to optimize cardiovascular status, reflecting that the current standard care renders patients under-resuscitated before, during and after surgery.

## Conclusion

We have shown an 8.9% rate of KD in patients undergone major orthopaedic procedures. We have highlighted the risk factors and showed the correlation thereof with the development of KD peri-operatively. We believe that close monitoring of this selective group of patients and limitation of use of nephrotoxic agents could lead to a significant decrease of KD in these cases. However, we acknowledge that larger patients sample is needed in order to identify other clinical and laboratory factors that may contribute to KD and thus improve our quality of everyday practice.

## Competing interests

The authors confirm that there are no conflicts of interest for the above manuscript.

## Authors’ contributions

KK, CD, GM, SAP and SG participated in the design of the study, data acquisition and analysis and writing of this manuscript. SG, KK and VS participated in the analysis and writing of this paper. VS participated in the analysis and also in revising critically the manuscript. All authors read and approved the final manuscript.

## Pre-publication history

The pre-publication history for this paper can be accessed here:

http://www.biomedcentral.com/1471-2369/13/101/prepub
